# Evaluation of the Physico-mechanical Properties and Electrostatic Charging Behavior of Different Capsule Types for Inhalation Under Distinct Environmental Conditions

**DOI:** 10.1208/s12249-020-01676-2

**Published:** 2020-05-12

**Authors:** Joana T. Pinto, Thomas Wutscher, Milica Stankovic-Brandl, Sarah Zellnitz, Stefano Biserni, Alberto Mercandelli, Mirjam Kobler, Francesca Buttini, Laura Andrade, Veronica Daza, Susana Ecenarro, Laura Canalejas, Amrit Paudel

**Affiliations:** 1 Research Center Pharmaceutical Engineering (RCPE) GmbH, Inffeldgasse 13, 8010 Graz, Austria; 2 Institute for Process and Particle Engineering, Graz University of Technology, Inffeldgasse 13/II, 8010 Graz, Austria; 3 MG2, Via del Savena 18, 40065 Pian di Macina di Pianoro, Bologna, Italy; 4 MEGGLE Excipients and Technology, Megglestraße 6-12, 83512 Wasserburg, Germany; 5 Food and Drug Department, University of Parma, Parco delle Scienze 27, 43121 Parma, Italy; 6 Laboratorios Liconsa, S.A. Avenida Miralcampo 7, 19200 Azuqueca de Henares, Guadalajara Spain; 7 Qualicaps, Avda. Monte Valdelatas 4, 28108 Alcobendas, Madrid, Spain

**Keywords:** gelatin, hydroxypropyl methylcellulose (HPMC), tribo-charging, water, dry powder inhaler (DPI)

## Abstract

Capsule-based dry powder inhaler (DPI) products can be influenced by a multitude of interacting factors, including electrostatic charging. Tribo-charging is a process of charge transfer impacted by various factors, *i.e.*, material surface characteristics, mechanical properties, processing parameters and environmental conditions. Consequently, this work aimed to assess how the charging behavior of capsules intended for inhalation might be influenced by environmental conditions. Capsules having different chemical compositions (gelatin and hydroxypropyl methylcellulose (HPMC)) and distinct inherent characteristics from manufacturing (thermally and cold-gelled) were exposed to various environmental conditions (11%, 22% and 51% RH). Their resulting properties were characterized and tribo-charging behavior was measured against stainless steel and PVC. It was observed that all capsule materials tended to charge to a higher extent when in contact with PVC. The tribo-charging of the thermally gelled HPMC capsules (Vcaps^®^ Plus) was more similar to the gelatin capsules (Quali-G™-I) than to their HPMC cold-gelled counterparts (Quali-V^®^-I). The sorption of water by the capsules at different relative humidities notably impacted their properties and tribo-charging behavior. Different interactions between the tested materials and water molecules were identified and are proposed to be the driver of distinct charging behaviors. Finally, we showed that depending on the capsule types, distinct environmental conditions are necessary to mitigate charging and assure optimal behavior of the capsules.

**Electronic supplementary material:**

The online version of this article (10.1208/s12249-020-01676-2) contains supplementary material, which is available to authorized users.

## INTRODUCTION

Dry powder inhalers (DPIs) deliver powder formulations containing active pharmaceutical ingredients (APIs) to the lungs through patients’ inhalation. They are mainly used in the treatment of respiratory diseases such as asthma, chronic obstructive pulmonary disease (COPD), and cystic fibrosis (36). The powder formulation is delivered using a device that allows its aerosolization and adequate delivery to the lung (15). DPI devices vary widely in design (7). Depending on whether the inhalers are breath-activated or not, these can be further categorized as passive or active devices, respectively (8). DPI devices contain either single or multi-doses. Multi-dose inhalers contain the powder stored as a bulk in a reservoir within the device to be metered prior to actuation, or alternatively, the powder is contained inside a series of pre-metered unit doses (blisters, capsules, tubes, dimples) that are delivered as single doses during each actuation (2,23). In a single dose inhaler, the patient has to load a pre-metered single dose of the powder into the device before each use (23). Pre-meter dosing is most commonly carried out using capsules (2,11,23).

Capsules used in DPIs are most usually composed of gelatin or hydroxypropyl methylcellulose (HPMC). Hard-gelatin capsules were the first ones to be introduced into the market (23). Briefly, gelatin capsules can be produced by immersing lubricated pins in a heated aqueous solution containing 30–40 wt% gelatin and a mixture of other materials (*e.g.*, colorants, surfactants) dependent on the proprietary formulation of each manufacturer (16,39). Once withdrawn from the solution, the pins are air-dried and after the completion of the film-forming process, the capsule shells are stripped from the pins using soft metal jars (16). HPMC capsules were later developed as an alternative to gelatin ones and they present certain advantages such as not being derived from animal sources, being more chemically inert, and retaining/adsorbing water to a lesser extent (1,28,39). HPMC capsules can be produced either by employing gelling agents or by using a thermal-gelling process. The first process is known as cold-gelling and was developed by Qualicaps (Quali-V®), using κ-carrageenan as a gelling agent and potassium chloride as a network promotor (28). The different manufacturing processes result in HPMC capsules with distinct properties. For instance, it has been demonstrated that thermally gelled HPMC capsules have smoother outer surfaces (39) and different mechanical properties (12) than their cold-gelled counterparts. Therefore, it is important to evaluate how capsule properties can be impacted by different inherent material attributes (gelatin *vs.* HPMC) and inherent characteristics from manufacturing (thermal-gelling *vs.* cold-gelling).

The handling of DPI products can be influenced by multiple factors (44), and tribo-charging is known to be one of them. Tribo-electrification, still not fully understood, is a process of charge transfer during frictional contact and subsequent separation of two solid surfaces (18,19,40). In insulator materials such as polymers, tribo-charging involves the exchange of electrons, ions, and material between two contacting surfaces, developing a positive or negative net charge. Therefore, the acquired net charge is a consequence of the contribution of each mechanism and results in the imbalance of positive and negative species (22,27). Tribo-charging depends on various factors, *i.e.*, material surface characteristics, mechanical properties, processing parameters, and environmental conditions like relative humidity (RH) (18). It is also often observed that capsules acquiring charge during the filling process rock and jump, increasing the number of rejects. Therefore, although tribo-charging can affect behavior of capsules for numerous applications, in the present work, the authors have focused on comparing different capsule types used for DPI. In the case of DPIs, tribo-charging of the capsules could also have a significant impact on the performance of the final product. Providing that charge can be transferred from the capsule to the fine powder contained inside, this can further lead to reduced delivered dose and reduced amount of the active substance in the lungs.

Therefore, this work aimed to assess how the charging behavior of capsules is influenced by their composition, inherent characteristics from manufacturing and environmental conditions, and how charge could potentially be mitigated in order to optimize handling of capsules. For this, hard-gelatin capsules (Quali-G^™^-I) and thermally and cold-gelled HPMC capsules (Vcaps^®^ Plus and Quali-V^®^-I, respectively) were stored at different relative humidities (RH). Their resulting properties (water content and mechanical properties) were characterized, and the charging behavior over stainless steel and PVC measured.

## MATERIAL AND METHODS

### Capsule Materials

This study was conducted with two-piece size 3 hard capsules used for inhalation. Gelatin (Quali-G^™^-I) and cold-gelled HPMC (Quali-V^®^-I) capsules were received from Qualicaps (Qualicaps Europe, S.A.U, Spain), and the thermally gelled HPMC capsules (Vcaps^®^ Plus) were received from Capsugel (a division of Lonza Group AG, Switzerland).

### Conditioning of the Capsule Materials

To equilibrate their moisture content with the gas phase prior to testing, all capsules were stored for at least 7 days in desiccators at different RH levels and RT (room temperature) of 22°C ± 2°C. Saturated solutions of lithium chloride, potassium acetate and magnesium nitrate were used to achieve a RH of 11%, 22% and 51%, respectively. Hygrometers were placed inside the desiccators to assure that the target RHs were achieved and maintained during the storage time.

### Characterization of the Capsule Material Charge

A GranuCharge™ (Granutools, Belgium) was used in a conditioned room (RH of 50% ± 3%, RT of 22°C ± 2°C) to measure the charging tendencies (*n* = 5) of the capsules stored at different RHs. The measurements were carried out in a similar manner to our previous work (45). Briefly, the initial density of charge (*q*0) of 1.2 g of capsules was evaluated by dispensing them into the in-built Faraday cup of the GranuCharge™. After that, the capsules were fed *via* a vibrational feeder into a V-tube and were dropped into the Faraday cup, where the density of charge was measured again (*q*1). V-tubes used in this study are tubes made of stainless steel or PVC, with an outer diameter of 5 cm, which are arranged at a 90° angle to each other and rotated so that they have an incline of 45° and an overall length of 70 cm. Before the measurements, it was ascertained that the operator and setup were grounded. The obtained charges were normalized by the mass of capsules dispensed into the apparatus, and the results are presented as charge to mass ratio (nC/g). The relative density of charge (Δ*q*) was obtained by subtracting the initial charge from the one acquired after contact with the V-tubes (*q*1 − *q*0). The tubes were cleaned with a dry paint roller and washed with isopropanol and let to dry completely between each analysis.

### Thermal Characterization of the Capsules

The capsules were characterized *via* modulated differential scanning calorimetry (MDSC). For this, small pieces were cut from the body of capsule and placed in an aluminum pan, ensuring that its base was properly covered (5.0 – 8.0 mg). The aluminum pans containing the samples were crimped using pierced lids and placed in a 204 F1 Phönix DSC (Netzsch, Germany), where the measurements were carried out at a nitrogen flow rate of 50 ml/min. Firstly, the samples were cooled from 20°C to − 40°C at a rate of 5°C/min and consequently heated from − 40°C to 225°C at a rate of 5°C/min using a modulation amplitude of 0.53°C every 40 s. The results were analyzed using Netzsch Proteus Thermal Analysis (Netzsch, Germany). The equipment is regularly calibrated for temperature and enthalpic response using indium.

### Mechanical Characterization of the Capsules

The mechanical properties of the capsules were measured at RT (22°C ± 2°C) and RH conditions (35% RH ± 3%) using an MCR compact Rheometer (MCR 300, Anton Paar, Austria). The capsules (*n* = 6) were placed centrally between two plates and compressed by the linear movement of the upper flat circular plate at a rate of 0.2 mm/s. The resulting normal force (N) and displacement (mm) values were recorded every 0.06 mm. The stress-strain curves were calculated from the recorded force-displacement profiles. The stress is considered to be the ratio of the applied load (normal force, N) by the cross section of the capsule that is expected to change during the test and could not be measured *in situ*. Therefore, a simplification was applied and the cross section was considered to be equal to the length of the capsules. The strain was determined by dividing the change in the depth (displacement) by the original diameter of the capsules (13). The elastic modulus was determined by linear regression from the slope of the elastic region of the stress-strain curves. The yield strength was considered to be the point at which the computed trend line crosses the stress-strain curves of the materials and plastic deformation begins.

### Water Content Determination

The water content of the gelatin and HPMC capsules (*n* = 3) was determined after 7 days of storage at 11%, 22% and 51% RH by Karl-Fischer titration. For the Karl-Fischer titration, Titroline 7500 KF was used (SI Analytics, Mainz, Germany) and the water was extracted from the capsules for 300 s in a mixture of CombiMethanol and dry formamide (1:1). The total water extracted from the capsules was quantified using Combititrant5 (Merck KGaA, Germany) as a titrant.

## RESULTS AND DISCUSSION

### Surface Chemistry of the Different Capsule Materials

**Fig. 1:**
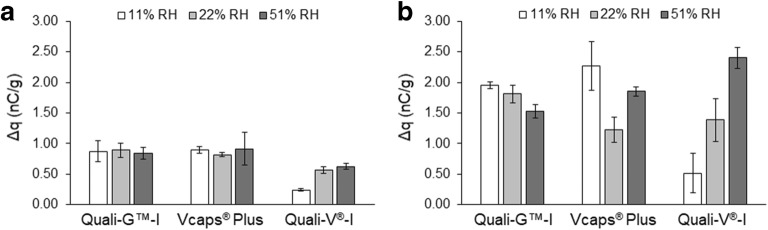
Relative density of charge after contact with stainless steel (**a**) and PVC (**b**) (*n* = 5, mean ± SD) of the capsules obtained after storage at different relative humidities (RHs)

To discuss how the surface chemistry of the distinct capsule materials could influence their tribo-charging behavior, the measurements that were carried out after storage at dry conditions (11% RH), where influence of water is expected to be minimal, are first considered. At 11% RH, the gelatin capsules (Quali-G^™^-I) and the thermally gelled HPMC capsules (Vcaps^®^ Plus) showed the highest charges when in contact with stainless steel (Fig. 1). The Vcaps^®^ Plus charged to the highest extent with PVC. On the contrary, the Quali-V^®^-I charged to the lowest extent when in contact with both stainless steel and PVC. In literature, it is reported that when in contact with an acrylic surface at various RHs, Quali-G^™^-I capsules always charged more than Quali-V^®^-I ones (37). However, as far as we know, this is the first study reporting the comparison of the charging behavior of HPMC capsules produced by two different processes. Here at low RH, Vcaps^®^ Plus capsules were found to charge to a higher extent. The behavior of the Vcaps^®^ Plus capsules showed to be more similar to the one observed for Quali-G™-I samples than that of Quali-V^®^-I.

Tribo-charging is an interfacial phenomenon and thus naturally dependent on the surface chemistry of the materials involved in the contact. Gelatin is a biopolymer consisting mainly of proteins (85 – 92%) (9). A study reports that flour powders with a higher content of the proteins tend to charge more positively when in contact with polytetrafluorethylene (PTFE) and PVC (35). Observation of Quali-G^™^-I charging on PVC surface (Fig. 1b) revealed a similar behavior, explaining the obtained results. Also, HPMC powders, cellulose ethers, have been shown to charge positively when in contact with stainless steel (14), similar to our results. However, as such, this does not explain why different HPMC capsules charged differently. While Vcaps^®^ Plus capsules are composed mainly of HPMC and trace water (39), Quali-V^®^ capsules also contain a trace amount of κ-carrageenan and potassium chloride (28). K-carrageenan is a polysaccharide containing sulfate groups that are conjugate bases, potentially able to accumulate hydrated protons in their vicinity (3). Therefore, we hypothesize that, similar to HPMC, κ-carrageenan could also contribute to the positive charging character of Quali-V^®^-I capsules. In contrast, potassium chloride (KCl) is known to charge negatively when in contact with glass and polystyrene (34), considering the position of glass in the tribo-electric series (4), it is expected that KCl also charges negatively against stainless steel and PVC. So, we postulate that the smaller positive charge observed for Quali-V^®^-I capsules could be a result of the shift in the overall net charge balance of Quali-V^®^-I capsules due to KCl. To map the chemical distribution within the outer capsule surface and its influence on the charge behavior, surface sensitive chemical profiling is necessary using techniques such as time-of-flight secondary ion mass spectrometry (ToF-SIMS) or X-ray photoelectron spectroscopy (XPS).

Different surface chemistries can also lead to distinct water sorption tendencies. The role of water in the properties of the different capsule materials (*i.e.*, tribo-charging behavior and mechanical properties) will be discussed in the next sections.

Although similar charging tendencies (*i.e.*, all samples charge positively) were found when the capsules were tested against stainless steel and PVC, it was clear that a higher extent of charge was acquired when measurements were carried out over PVC. Observation of the tribo-electric series reveals that PVC is a more electro-negative material than stainless steel (10), so capsules will evidently charge more positively when in contact with the polymer.

Finally, considering that capsule-capsule contacts may as well influence the tribo-charging, an initial study was also performed to test those interactions. The latter were simulated by covering the inside of the V-tubes with foils made of the capsule material. It was observed that although there was some charge transfer in the negative range, this was too low to be quantified. Therefore, the contact charges seen in this study are in their majority the result of the interactions of the capsules with the PVC and stainless-steel surfaces.

### Surface Topography and Mechanical Properties of the Different Capsule Materials

Under a given applied load, the magnitude of a real contact between two materials will be determined by their deformation properties and surface topography (6). As tribo-charging is a contact-driven phenomenon, it will evidently be influenced by the mechanical properties of materials in contact. For instance, Wauthoz *et al*. have shown that the outer shell of Quali-V^®^-I capsules is less uniform than that of Vcaps^®^ and Quali-G^™^-I capsules (39). Investigation into the influence of surface topography (*i.e.*, presence of valley, peaks, or clefts of varying length scales on the material surface) on tribo-charging has shown that an increase in the mean micron-sized roughness at the surface of acrylic polymer powders led to a decrease in charge density. The authors attributed the former to the existence of fewer contact points available for charge transfer (38). Also, the impact of roughness surface distribution has been studied and has shown that when the distance between two peaks increases, materials charge more homogenously (29). In this way, the relatively rougher surface of the Quali-V^®^-I capsules could provide another explanation for the given results, *i.e.*, why, generally, these materials charged to a lesser extent.

**Fig. 2:**
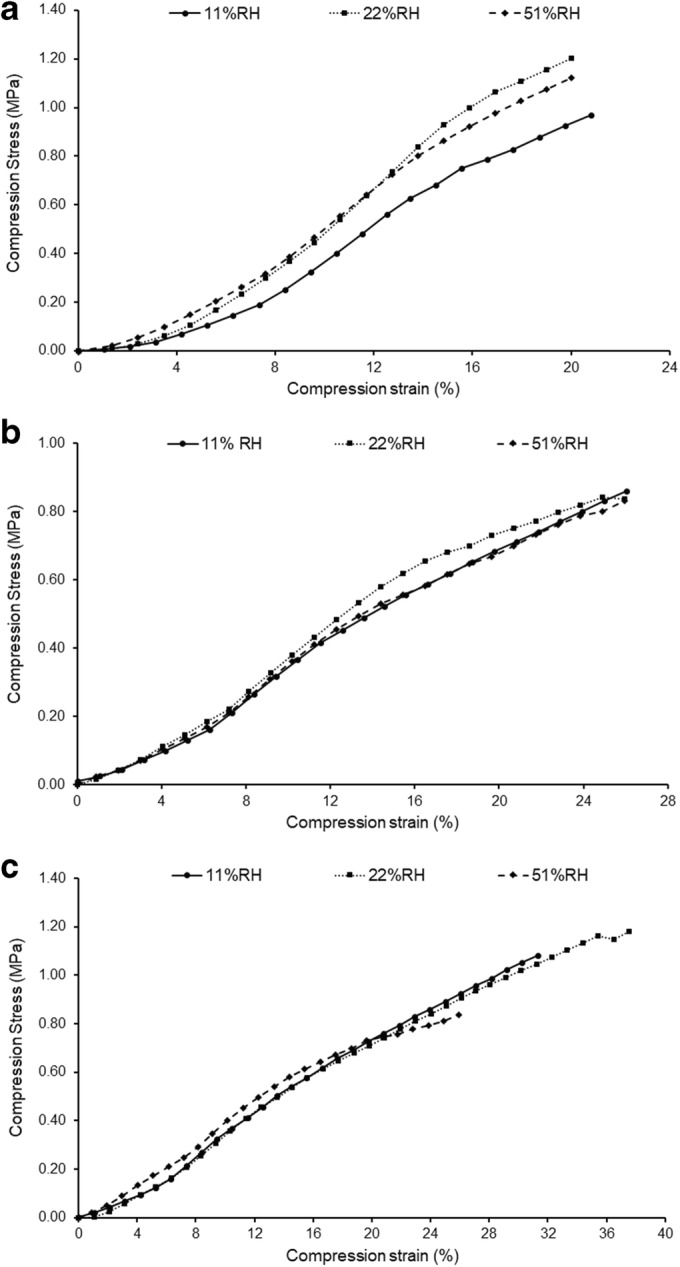
Average result (*n* = 6) of the stress-strain curves of the different capsules: Quali-G^™^-I (**a**), Vcaps^®^ Plus (**b**), and Quali-V^®^-I (**c**) obtained after storage at different relative humidities (RHs)

Bulk mechanical properties of the capsules were characterized, and the resulting stress-strain profiles are presented in Fig. 2. The derived mechanical parameters from the curves are listed in Table I. Contacts between two surfaces can be elastic or plastic, and the real area of contact inversely correlated to the elastic modulus and hardness of the materials, respectively (6). In line with a previous work using the same methodology to test the mechanical properties of capsules (26), we showed that gelatin capsules have a higher elastic modulus than HPMC capsules and present almost no plastic deformation. This indicated that primarily Quali-G^™^-I capsules deform elastically (42) and HPMC ones elastically and plastically (12,26) (since HPMC is a typical viscoelastic polymer). Given the different modes of deformation, the inherent mechanical properties of these two polymers can originate different extents of impaction and friction during the tribo-electrification process. However, observation of the mechanical properties of the HPMC capsules after storage at 11% RH shows that these properties are similar (Table I). Thus, it seems that in the dry state, the contribution of other properties prevails (*i.e.*, surface chemistry and roughness) in driving the observed charging differences between the Vcaps^®^ Plus and Quali-V^®^-I.

**Table I:** Bulk Mechanical Properties of the Capsule Materials (*n* = 6, Mean ± SD) Measured After Storage at Different Relative Humidities (RHs)

Sample	Elastic modulus (MPa)			Yield strength (MPa)		
	11% RH	22% RH	51% RH	11% RH	22% RH	51% RH
Quali-G^™^-I	5.27 ± 0.44	6.91 ± 0.36	6.12 ± 0.57	0.51 ± 0.04	0.67 ± 0.05	0.57 ± 0.05
Vcaps^®^ Plus	3.73 ± 0.48	4.11 ± 0.52	3.70 ± 0.33	0.64 ± 0.07	0.65 ± 0.07	0.57 ± 0.19
Quali-V^®^-I	3.94 ± 0.43	3.43 ± 0.08	3.99 ± 0.38	0.72 ± 0.06	1.12 ± 0.27	0.66 ± 0.08

### Impact of Conditioning on the Thermo-physical Properties of the Different Capsule Materials

**Fig. 3:**
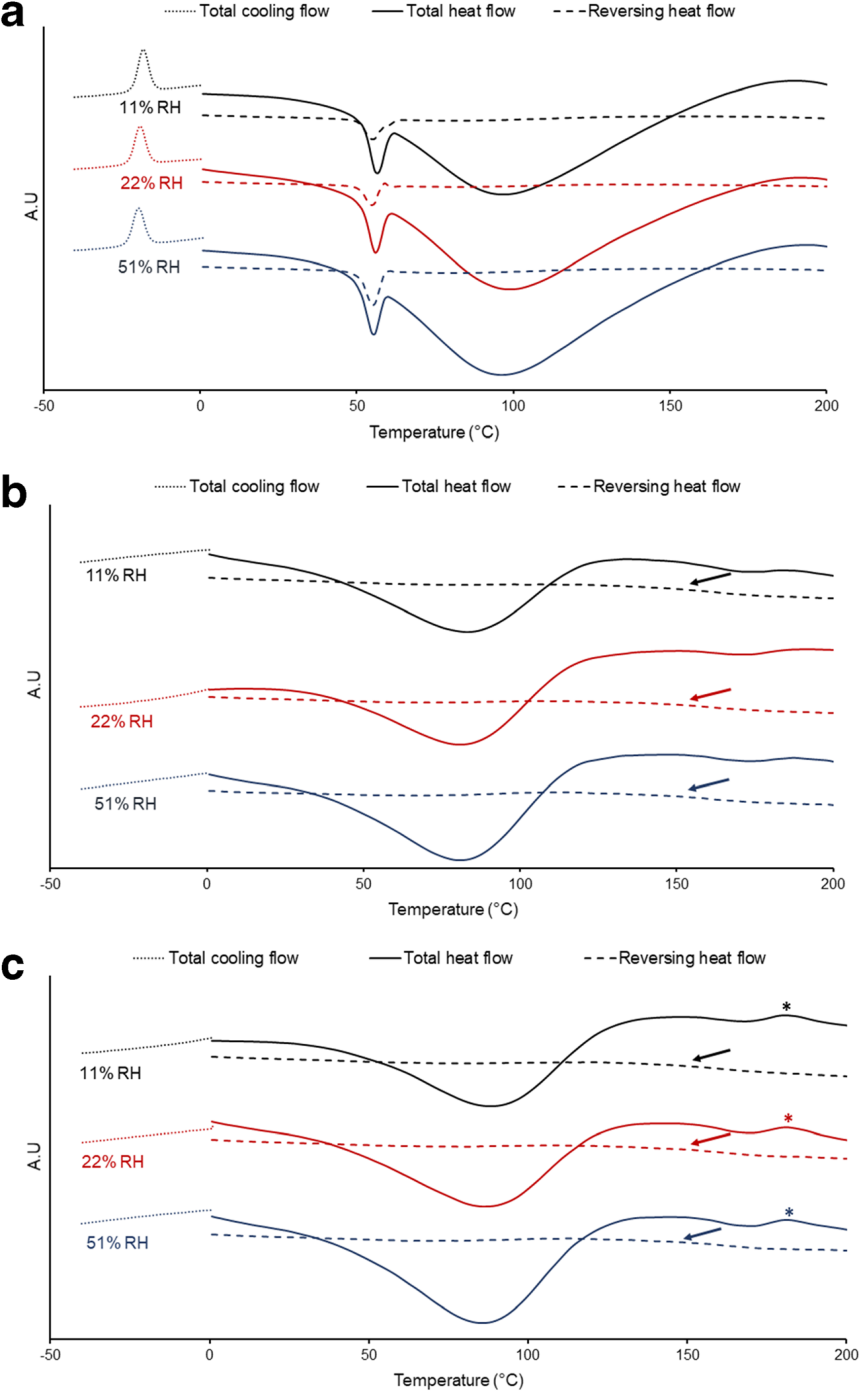
Representative thermograms of the different capsule materials: Quali-G™-I (**a**), Vcaps^®^ Plus (**b**), and Quali-V^®^-I (**c**) obtained after storage at different relative humidities (RHs). The arrows point to the glass transition events identified for Vcaps^®^ Plus and Quali-V^®^-I, and the asterisk points to exothermic events detected for Quali-V^®^-I (see Supplementary Material for better visualization)

The impact of different RHs on the solid state of the different capsule materials was evaluated using MDSC. After conditioning at all the selected RHs, thermal analysis of the Quali-G^™^-I capsules revealed an exothermic event during cooling of the samples (Fig. 3). The exotherm was attributed to freezing of the free and loosely bound water molecules (30). At about 50°C, a sharp endotherm was detected. We hypothesized that this could be due to conformational changes in the protein structure of gelatin (25). Over time, it was possible to observe a decrease in the onset temperature of the exothermic event (Table II), potentially due to the increase in the fraction of loosely bound water (in relation to the pure free fraction). Interestingly, a decrease in the melting endotherm at 50°C was simultaneously identified. We propose that this could have been the result of a difference in the fraction of loosely bound water molecules in the protein structures of gelatin. For Quali-G^™^-I, a large endotherm was also identified due to the onset of water evaporation at about 65–71°C. No identifiable change in the enthalpy of water evaporation could be detected after conditioning at the different RHs. Finally, the glass transition event of commercial gelatin reported to be at about 40–70°C (31) was not possible to be detected in this work. For the Vcaps^®^ Plus, a clear glass transition event was detected at about 145°C (Fig. 3). It has been reported that HPMC containing low water contents (see Table III) have a glass transition temperature around 145°C (47); therefore, the detected event is attributed to HPMC. A slight decrease in the onset of the glass transition was noticed after conditioning at 51% RH, indicating some plasticization of the material at higher RHs. An endotherm due to water evaporation was detected at about 40°C, and the enthalpy of the event increased (Table II) as the Vcaps^®^ Plus samples were conditioned at higher RHs. Lastly, thermal analysis of the Quali-V^®^-I samples also revealed a glass transition event at about 145°C. However, contrary to their Vcaps^®^ Plus counterparts, no changes in the event could be detected when the samples were stored under different RHs. For the Quali-V^®^-I, an additional exotherm was detected at 172°C (Fig. 3). The interaction of potassium cations with sulfate esters of κ-carrageenan has been shown to influence the thermal behavior of the polymer (32), pointing to a potential explanation for the event detected here. However, this needs further verification. Notwithstanding, the exothermic event was not affected by the conditioning under different RHs. Finally, an endotherm due to the evaporation of water onset at about 40°C was also detected for Quali-V^®^-I. An increase in the enthalpy of the evaporation of water (Table II) was only detected after conditioning of the Quali-V^®^-I capsules at 51% RH.

**Table II:** Thermo-physical Properties of Capsules Obtained *via* MDSC Analysis (*n* = 3, Mean ± SD) After Storage at Different Relative Humidities (RHs)

Sample	Quali-G™-I		
MDSC results	Exotherm *T*_onset_ (°C) / enthalpy (J/g)	Melting endotherm *T*_onset_ (°C) / enthalpy (J/g)	Water evaporation endotherm *T*_onset_ (°C) / enthalpy (J/g)
11% RH	−21.80 ± 0.17 / 7.88 ± 1.20	52.33 ± 0.49 / 10.59 ± 1.07	71.20 ± 0.30 / 204.17 ± 35.69
22% RH	−22.67 ± 0.40 / 6.90 ± 0.48	51.53 ± 0.55 / 9.44 ± 0.33	70.57 ± 1.12 / 209.87 ± 11.00
51% RH	−23.37 ± 0.25 / 6.81 ± 0.11	50.63 ± 0.68 / 8.95 ± 0.32	65.73 ± 2.92 / 208.13 ± 1.42
Sample	Vcaps® Plus		
MDSC results	Water evaporation endotherm *T*_onset_ (°C) /enthalpy (J/g)	Glass transition *T*_onset_ (°C) /heat capacity (J/g· K)	
11% RH	41.40 ± 2.10 / 86.67 ± 13.93	143.10 ± 4.95 / 0.09 ± 0.02	
22% RH	43.40 ± 2.36 / 92.40 ± 11.12	146.13 ± 0.46 / 0.10 ± 0.05	
51% RH	42.87 ± 0.64 / 109.40 ± 5.04	136.83 ± 4.71 / 0.15 ± 0.05	
Sample	Quali®-V I		
MDSC results	Water evaporation endotherm *T*_onset_ (°C) /enthalpy (J/g)	Glass transition *T*_onset_ (°C) / heat capacity (J/g· K)	Exotherm *T*_onset_ (°C) / enthalpy (J/g)
11% RH	47.67 ± 3.23 / 107.93 ± 12.04	141.90 ± 4.36 / 0.12 ± 0.03	172.47 ± 0.64 / 3.05 ± 0.29
22% RH	41.60 ± 0.92 / 106.10 ± 3.16	145.87 ± 0.80 / 0.10 ± 0.04	171.90 ± 0.30 / 3.46 ± 0.25
51% RH	42.67 ± 1.60 / 171.93 ± 0.35	142.40 ± 4.66 / 0.11 ± 0.04	171.93 ± 0.35 / 3.10 ± 0.32

*MDSC* modulated differential scanning calorimetry

**Table III:** Total Water Content Measured *via* Karl-Fischer Titration (*n* = 3, Mean ± SD)

Sample	Water content (wt%) of capsules after storage at		
	11% RH	22% RH	51% RH
Quali-G™-I	7.18 ± 1.29	11.25 ± 0.69	13.62 ± 0.92
Vcaps^®^ Plus	2.41 ± 0.30	3.63 ± 0.03	6.68 ± 0.68
Quali-V^®^-I	2.17 ± 0.15	3.44 ± 0.16	6.02 ± 0.70

*RH* relative humidity

### Impact of Conditioning on the Mechanical Properties

Conditioning at different RHs influenced the mechanical properties of the materials (Table I), probably due to the different extents of water sorption (Table III) and interactions of the water molecules with gelatin and HPMC (shown by MDSC). The elastic modulus of the Quali-G™-I capsules increased at 22% RH (11.25 ± 0.69 wt% of water) and decreased again at 51% RH (13.62 ± 0.92 wt% of water). These observations were in agreement with other reported work (42) and are probably the consequence of the change in the amount of water molecules bound to the helical fragments of proteins in gelatin (as also observed by MDSC). The transient increase in the elastic modulus and yield strength from the dried samples to storage at 22% RH could potentially be explained by gelatin densification occurring between 0 and 11.8 wt% water content (42). After conditioning at 51% RH, the elastic modulus and yield strength decreased once again. The conditioning of the Vcaps^®^ Plus capsules at higher RH (51% RH) slightly decreased the yield strength, possibly due to plasticization (12,30) (supported by the decrease in Tg observed *via* MDSC). However, at 22% RH, a transient increase of the elastic modulus was observed. In previous works, similar observations have been reported when starch-based films were plasticized with glycerol (5,24,46). This phenomenon was proposed to be due to the β-relaxation temperature (not detected by MDSC) of the system being higher than the β-relaxation of the pure starch and α-relaxation (Tg) of glycerol. The latter counter-intuitive observation is proposed to be the result of a strong molecular interaction between the starch and the glycerol, resulting in an increase of the β-relaxation of the polymer-plasticizer system (24). Interestingly, in a previous work, the authors have identified a β-relaxation event for thermally gelled HPMC capsules under dry conditions that almost disappeared after storage at 50% RH (12). For the Quali-V^®^-I capsules, the uptake of water did not result in higher elastic modulus material than the one reached when stored at 22% RH. However, a decrease of the yield strength was also observed at 51% RH, indicating the potential plasticization of the material (however, no decrease in Tg could be observed *via* MDSC).

### Impact of Water Sorption on Charge

Charge evolution upon storage at the different RHs revealed distinct tendencies for different materials (Fig. 1). While almost no differences could be observed in the charging behavior over stainless steel, all the capsules charged more over PVC. Clear statistically significant (see Supplementary Material) trends could be identified at the different RHs used for conditioning the distinct capsule materials before these were tested against PVC. Conditioning of the Quali-G™-I capsules at higher RH decreased the charging over the polymer. For the Vcaps^®^ Plus capsules, charge decreased with increased humidity from 11 to 22% RH but increased once again when the humidity reached 51%. The Quali-V^®^-I capsules showed a systematic increase of charge with storage RH. As previously documented (21,28), gelatin capsules contain more water than HPMC ones (Table III). At 11% RH and 22% RH, the Quali-G™-I capsules contain 3 times more water than the HPMC capsules. Both HPMC capsules showed similar water contents at all the tested RHs, taking a substantial amount of water at higher humidities (from about 3.5 to 6.0 wt%). Considering the overall similar water content of the 2 HPMC capsule types and their dissimilar tribo-charging, it is proposed that the specific interaction of water molecules with the different capsule films mainly results into the distinct tribo-charging behaviors.

For the gelatin capsule, literature indicates that at 20% RH, a complete monomolecular layer of water is formed (43) and that conditioning at increasing humidities lead to water being mainly condensed onto the surface of the capsules and only very small percentage is absorbed into the material (43). In the case of HPMC, water adsorption onto its surfaces has been shown to result mainly in its absorption into the material (30). Indeed, this was confirmed by MDSC, by which free/loosely bound molecules of water were only identified for the Quali-G™-I samples. Investigations of the charging behavior of polymers at different RHs have shown that tribo-charging is dependent on the nature of the material and in particular on the formation of a water monolayer around it (33). At lower RHs, before a full monolayer is formed, water molecules present on the polymer surface seem to induce charge (20,33). However, at a critical RH, where a monolayer is formed, the authors suggest that a conducting hull is created around the surface and cross-over to smaller charging values is attained (33). Likewise, we speculate that for the gelatin capsules, the presence of free/loosely bound water results in the decrease of the charge observed with the increase in humidity. In contrast, for HPMC, the sorbed water interacts with the polymer and free/loosely bound water is not available to conduct the charge.

For the Quali-V^®^-I capsules, a systematic increase in charging with increase in RH was observed; for their Vcaps^®^ Plus counterparts, the charge first increased and then decreased. We hypothesize that these differences were due to the distinct ways that water might have interacted with the polymer. For the Vcaps^®^ Plus capsules, the initial increase in water lowered charging, potentially due to a strong interaction of water with the polymer (indicated by the mechanical properties at 22% RH), having led to a transient structural change that could have temporarily impacted species exchange during tribo-charging. However, as more water molecules are sorbed, it is hypothesized that water molecules will not bind exclusively to the polymer but interact with each other instead (41), potentially driving tribo-charging at 51% RH (20). For the Quali-V^®^-I capsules, at first, it is assumed that the water molecules interact with KCl (instead of the polymer) (17), shifting ion exchange between the contacting material and the Quali-V^®^-I samples (that is hypothesized to result in the negative charging of the polymer—see “Surface Chemistry of the Different Capsule Materials”) and explaining why a lower charge was observed. However, as more water is sorbed, the potential presence of a higher number of water molecules can result in the mediation of tribo-charging, resulting in greater charge of the material at 22% and 51% RH. In general, the water interaction with the polymer surfaces seems to be more important in the case of tribo-charging over PVC than over stainless steel. We propose that this might be due to water playing a more important role in case of insulator-insulator contacts than insulator-metal contacts (20). Indeed, the only process that seems to notably influence tribo-charging with stainless steel was the potential interaction of water molecules with the KCl present on the Quali-V^®^-I capsules.

### Practical Implications of the Study

Considering the detrimental role that tribo-charging can play in drug product manufacturing, the outcome of the present study provides a direction on how to distinguish the electrostatic charging of different capsule types intended for dry powder inhalation. It was verified that all the capsule materials charged less when in contact with stainless steel instead of a polymer material such as PVC. Although stainless-steel equipment is generally involved during encapsulation of pharmaceuticals, it is important to consider that certain machines can have small parts made of polymeric materials. Consequently, a careful consideration of the relative humidity must be taken into account and depending on the capsule material, different humidity conditions might be necessary to mitigate tribo-charging. In this study, we present information on how one can selectively choose the most adequate environmental settings to optimize the handling of pharmaceutical capsules intended for DPI. In our following communications, we will attempt to show how the in-process tribo-charging behavior of diverse capsule materials under different processing conditions and RHs can affect the *in vitro* performance of the capsule based DPIs. In general, we propose that the information obtained in the current study can be equally applicable to the handling of capsules intended for oral drug delivery.

## CONCLUSIONS

Tribo-charging can have a detrimental impact on the handling of pharmaceutical materials and can affect the aerodynamic performance and thus the pulmonary delivery of the API. In this study, the tribo-charging of capsules intended for dry powder inhalation was related to their inherent chemical composition, manufacturing process, and environmental humidity. We have shown that HPMC capsules manufactured using two different processes (thermally and cold-gelled) result in distinct tribo-charging behaviors. The Vcaps^®^ Plus capsules charged more after storage at lower humidities; in turn, Quali-V^®^-I samples were found to charge to a higher extent after conditioning at higher RHs. All capsules tended to charge to a higher extent in contact with PVC. The thermally gelled HPMC capsules charged more similarly to Quali-G™-I samples than to their HPMC Quali-V^®^-I counterparts. The sorption of water by the capsules notably impacted their mechanical properties and tribo-charging behavior. Different interactions between the polymers and water molecules were proposed to be the source of dissimilar tribo-charging behaviors, further evidencing the complex role of water in the tribo-charging of insulator materials. Finally, we showed that distinct environmental conditions might be necessary to control tribo-charging and ensure the optimal behavior (for *e.g.*, during processability) of different pharmaceutical capsule materials.

## Electronic Supplementary Material

ESM 1 (DOCX 2190 kb)

## Abbreviations

API: Active pharmaceutical ingredient
COPD: Chronic obstructive pulmonary disease
DPI: Dry powder inhaler
HPMC: Hydroxypropyl methylcellulose
KCl: Potassium chloride
PTFE: Polytetrafluoroethylene
PVC: Polyvinyl chloride
RH: Relative humidity
RT: Room temperature
SD: Standard deviation
Tg: Glass transition temperature
ToF-SIMS: Time-of-flight secondary ion mass spectrometry
XPS: X-ray photoelectron spectroscopy
