# Supplementary material for: Evaluation of the Physico-mechanical Properties and Electrostatic Charging Behavior of Different Capsule Types for Inhalation Under Distinct Environmental Conditions
Source: AAPS PharmSciTech. 2020 May 12;21(4):128. doi: 10.1208/s12249-020-01676-2 (PMC7217808; doi:10.1208/s12249-020-01676-2)
Supplement: Supplementary file 1 — (DOCX 2190 kb) [file 12249_2020_1676_MOESM1_ESM.docx]

**Supplementary Material**

**
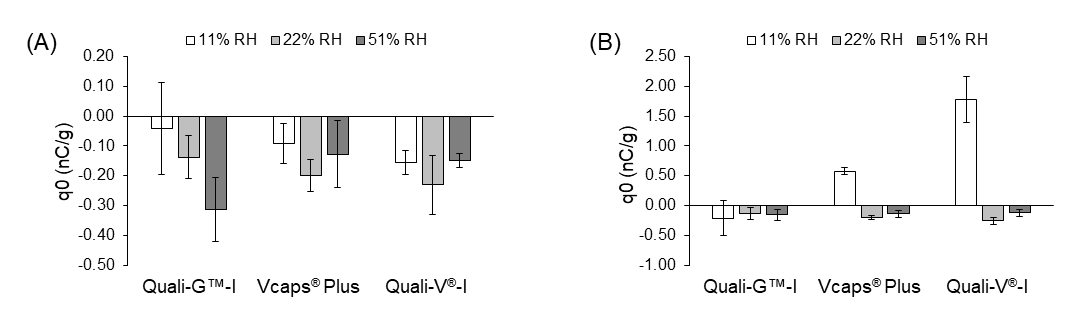
**

**Figure S1:** Initial charge of the capsules (q0) after storage at different relative humidities (RHs) and before coming into contact with stainless-steel (A) and PVC (B) (n=5, mean ± SD).


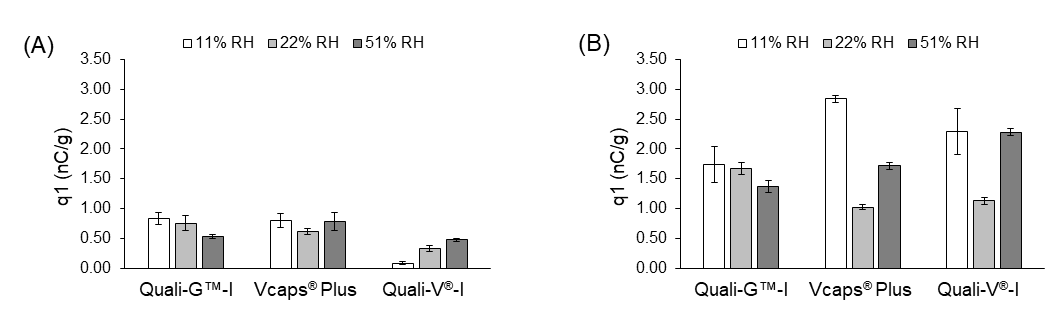


**Figure S2:** Charge of the capsules after storage at different relative humidities (RHs) and after coming contact (q1) with stainless-steel (A) and PVC (B) (n=5, mean ± SD).


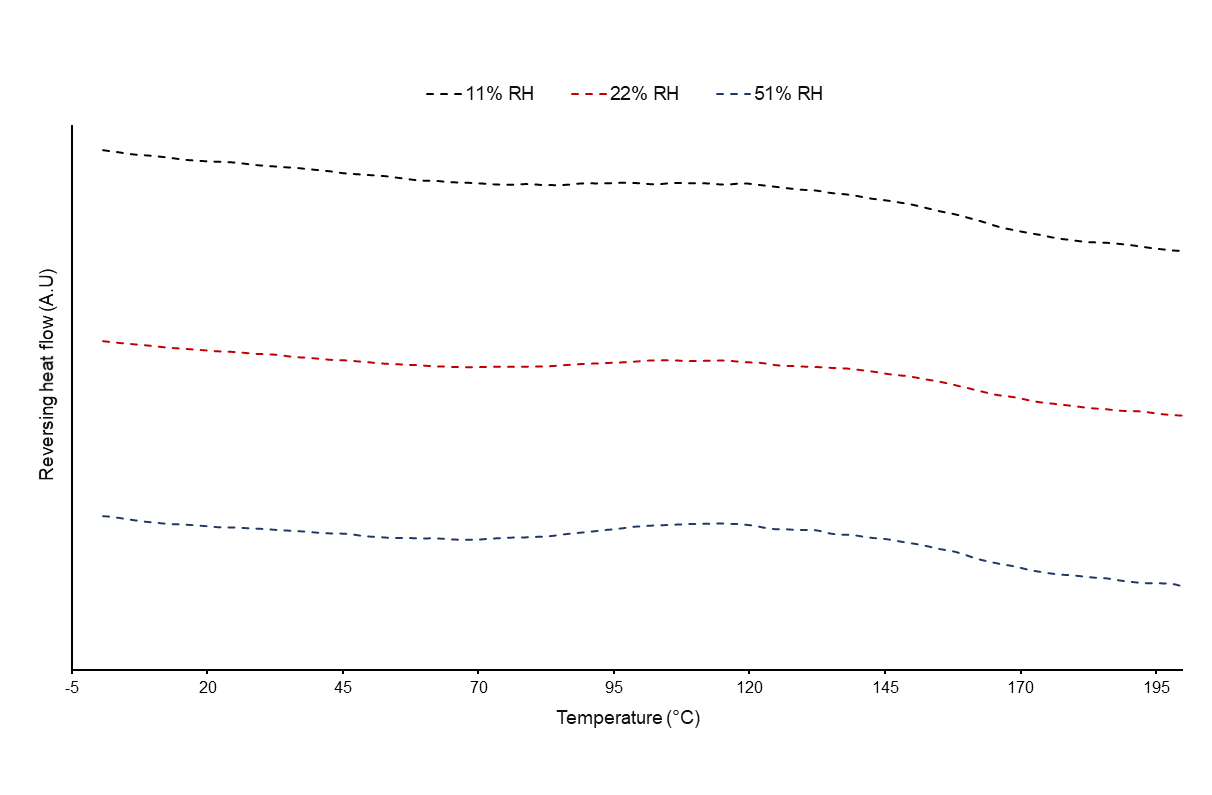


**Figure S3:** Representative thermograms showing the reversing heat flow curves of the Vcaps® Plus samples after conditioning at different relative humidities (RHs).

**
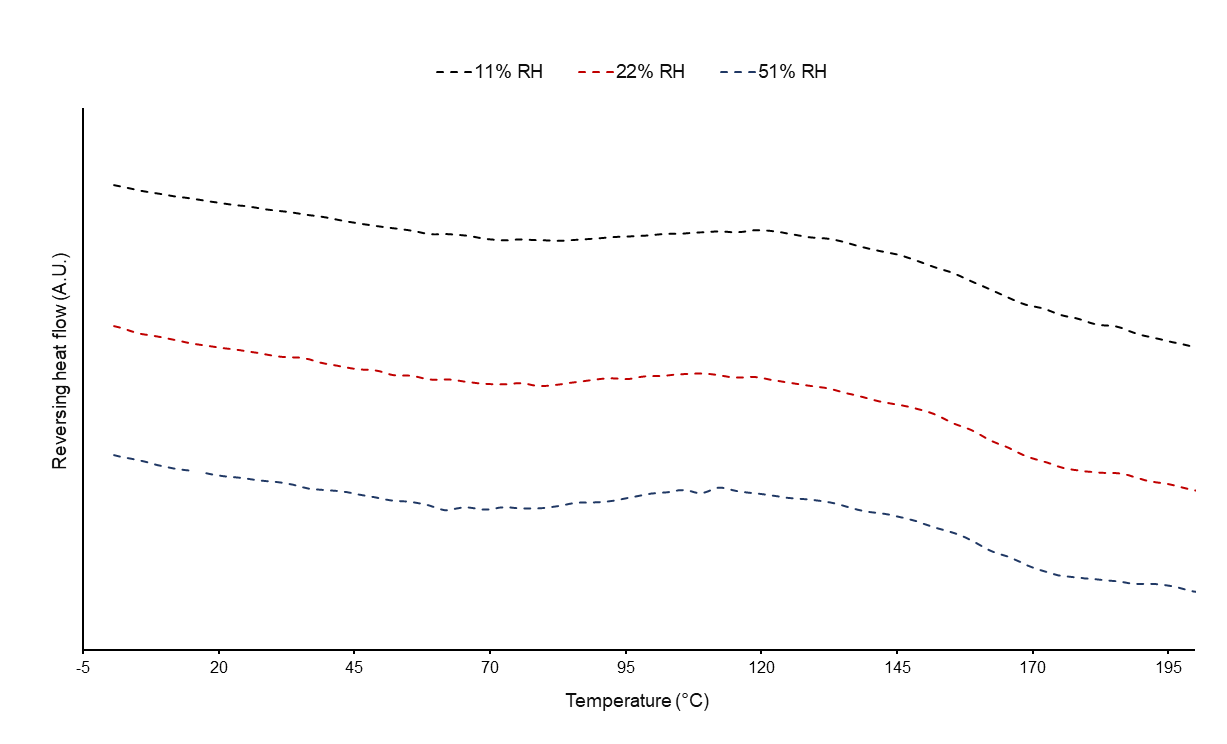
**

**Figure S4:** Representative thermograms showing the reversing heat flow curves of the Quali-V I samples after conditioning at different relative humidities (RHs).

**
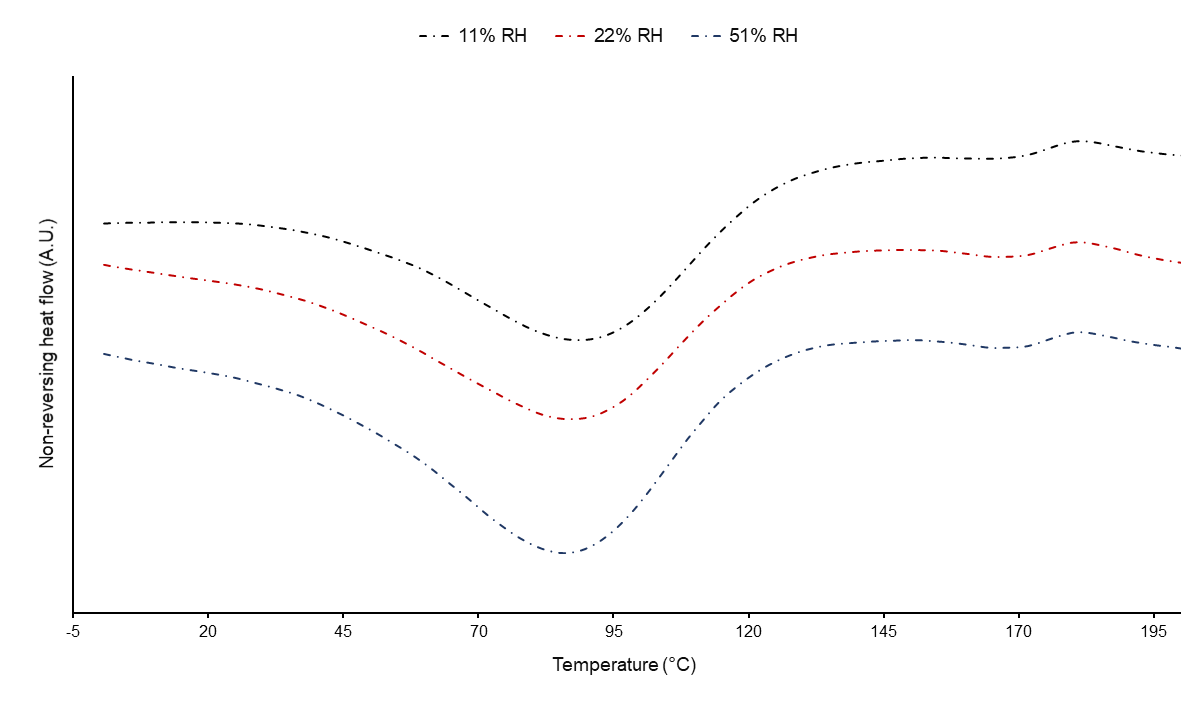
**

**Figure S5:** Representative thermograms showing the non-reversing heat flow curves of the Quali-V I samples after conditioning at different relative humidities (RHs).


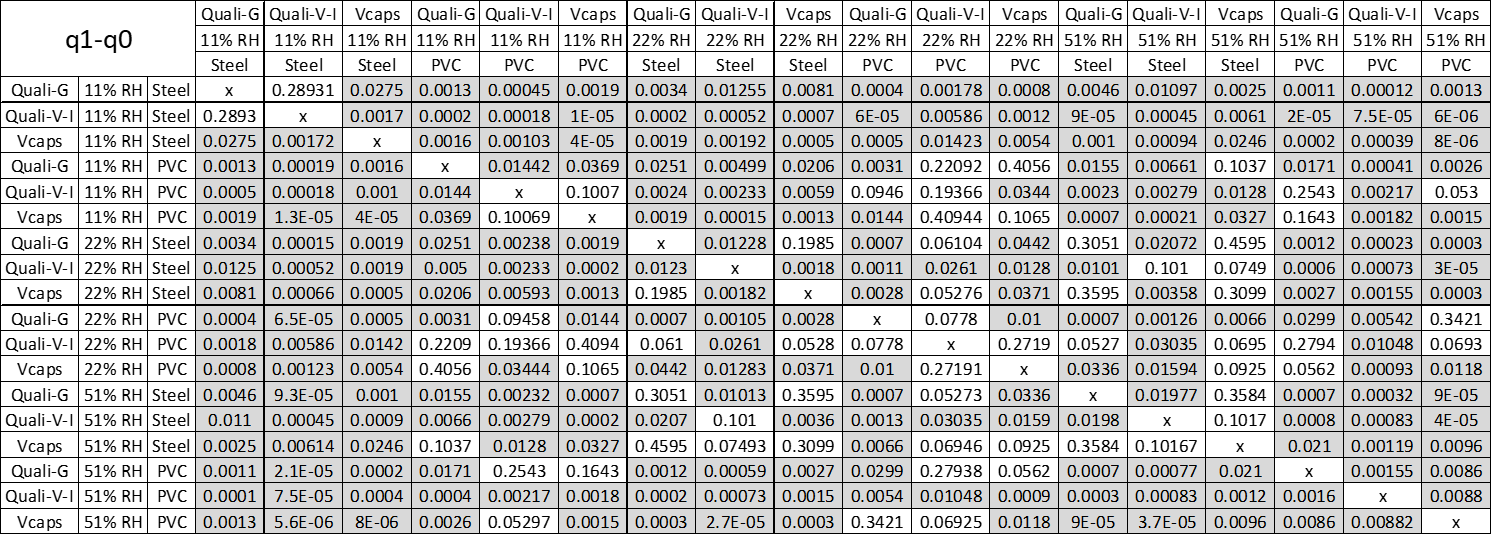


**Figure S6:** Statistical analysis (two sample t-test assuming unequal variances) of the density of charge of the different capsule materials conditioned under distinct relative humidities and tested against stainless-steel and PVC.
